# Modulation of Abiotic Stress Responses in Rice by E3-Ubiquitin Ligases: A Promising Way to Develop Stress-Tolerant Crops

**DOI:** 10.3389/fpls.2021.640193

**Published:** 2021-03-23

**Authors:** Fredilson Veiga Melo, M. Margarida Oliveira, Nelson J. M. Saibo, Tiago Filipe Lourenço

**Affiliations:** Instituto de Tecnologia Química e Biológica António Xavier, Universidade NOVA de Lisboa, Oeiras, Portugal

**Keywords:** ubiquitination, drought, salinity, cold, heat, proteasome

## Abstract

Plants are unable to physically escape environmental constraints and have, therefore, evolved a range of molecular and physiological mechanisms to maximize survival in an ever-changing environment. Among these, the post-translational modification of ubiquitination has emerged as an important mechanism to understand and improve the stress response. The ubiquitination of a given protein can change its abundance (through degradation), alter its localization, or even modulate its activity. Hence, ubiquitination increases the plasticity of the plant proteome in response to different environmental cues and can contribute to improve stress tolerance. Although ubiquitination is mediated by different enzymes, in this review, we focus on the importance of E3-ubiquitin ligases, which interact with the target proteins and are, therefore, highly associated with the mechanism specificity. We discuss their involvement in abiotic stress response and place them as putative candidates for ubiquitination-based development of stress-tolerant crops. This review covers recent developments in this field using rice as a reference for crops, highlighting the questions still unanswered.

## Introduction

Plants, as sessile organisms, have evolved myriad complex and efficient molecular and physiological mechanisms to cope with the various environmental constraints that affect their growth and development (Ahmad and Prasad, 2012). One such mechanism is the post-translational protein modification (PTM) ubiquitination. Ubiquitination is involved in virtually all cellular processes in eukaryotes, and it allows a fast remodel of target protein abundance. It is the core of the main pathway for degradation of proteins, the ubiquitin-proteasome system (UPS) (Hershko and Ciechanover, 1998). This PTM refers to the covalent attachment of the ubiquitously expressed 76 residues, 8 kDa protein, ubiquitin, to a lysine residue of a substrate protein, modifying its abundance, activity, or localization (Lee and Kim, 2011). Ubiquitination is also a reversible process. The fate of ubiquitin-targeted proteins can be reversed by the action of deubiquitinating enzymes (deubiquitinases, DUBs). These enzymes are ubiquitin-specific proteases, and their action is important not only to maintain the ubiquitin pool stability, but also to fine-tune the ubiquitination of proteins associated with different cellular processes (Nijman et al., 2005; March and Farrona, 2018).

In the most classical pathway, ubiquitination comprises a multistep process involving three enzymes operating in an ATP-dependent manner. First, the ubiquitin protein is activated by the E1-activase, subsequently transferred to the E2-conjugase, and finally the E3-ligase mediates the transfer of the ubiquitin to the target protein forming a bond between the glycine of the ubiquitin molecule and a lysine on the target protein (Yu et al., 2016). There are different types of ubiquitination: a single ubiquitin molecule can be attached to the target protein at a single amino acid (a.a.) residue (monoubiquitination) or multiple a.a. residues (multimonoubiquitination), modifying the protein’s function or location. In addition, ubiquitin can be attached to the target protein as a ubiquitin chain (polyubiquitination). Polyubiquitination is the most frequent type of ubiquitin modification, targeting a range of proteins, including abnormal proteins, receptors, and short-life regulatory proteins (Stone et al., 2005). These polyubiquitin chains can be assembled using the different lysine (K) residues present in the ubiquitin protein (K6, K11, K27, K29, K31, K48, and K63), thus driving the targeted protein to different fates, including degradation (mainly K48-linked chain) by the UPS (Lee and Kim, 2011; Zhang et al., 2015). The different types of polyubiquitin chains generate a ubiquitin code that increases the functional plasticity of this system beyond the regulation of protein degradation through the UPS. Among those, the K63-linked chains are implicated in the degradation of proteins and cellular compartments through endocytosis and selective autophagy (Clague and Urbé, 2010; Romero-Barrios and Vert, 2018; Su et al., 2020).

Among the ubiquitination enzymes, E3-ubiquitin ligases are the most abundant. The Arabidopsis, rice, and maize genomes are predicted to harbor more than 1100 genes encoding E3-ubiquitin ligases (Smalle and Vierstra, 2004; Du et al., 2009). Their abundance is thought to be related to their specificity for the target proteins (Vierstra, 2012). The E3-ubiquitin ligases can be divided into three main monomeric types: really interesting new gene (RING) type, homology to E6-associated carboxyl-terminus (HECT) type, and U-box type. These are differentiated by their structure and ability to form an intermediate during ubiquitination. The RING type is further divided into monomeric E3s or multi-subunit E3s [also known as Cullin-RING Ligases (CRLs)], depending on whether the E2- and substrate-binding functions are found in the same or different proteins, respectively (Deshaies and Joazeiro, 2009; Lyzenga and Stone, 2012). The CRLs are composed of several protein subunits, and the target protein specificity is given by a group of substrate-binding proteins [F-box, DDB1 binding WD40 (DWD) and broad complex tramtrack bric-a-brac (BTB)] (Lyzenga and Stone, 2012). Interestingly, the CRL family is shown to be involved in signaling regulation of the major classes of phytohormones (auxins, jasmonates, gibberellins, etc.) (Kelley and Estelle, 2012). Further insights into the mechanisms and chemistry of ubiquitination in plant systems are extensively reviewed elsewhere (Vierstra, 2009, 2012; Callis, 2014).

Over the last decade, ubiquitination has emerged as a target for the improvement of crop stress tolerance (Dametto et al., 2015; Xu and Xue, 2019). Most of the studies regarding the ubiquitination pathway in plant responses to environmental stresses have focused on monomeric E3-ubiquitin ligases, due to their abundance and specificity, and their role in different biotic and abiotic stresses (e.g.: drought, salinity, radiation, and nutrient deprivation). In these studies, E3-ubiquitin ligases are associated with the regulation of phytohormones, protein stability, and levels of heavy metals to mention only a few (Dametto et al., 2015). The large number of different E3-ubiquitin ligases (with different structure, subcellular localization, and so on) suggest a high level of versatility and a possible role in a multitude of cellular processes (Stone et al., 2005). However, the regulation of abiotic stress responses by E3-ubiquitin ligases remains largely elusive as well as most of their target proteins.

**FIGURE 1:**
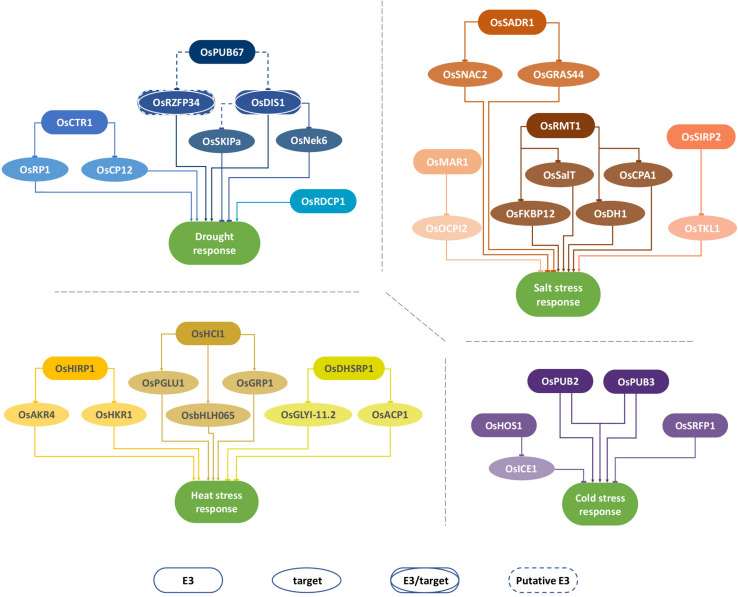
Selected E3-ubiquitin ligases involved in abiotic stress response in rice. Illustration of rice E3-ubiquitin ligases involved in plant response to drought, salt, heat, and cold stress. The illustration displays selected E3 ligases and their target proteins when available. Dashed lines denote known interaction but unknown effect over the target protein, namely whether it is targeted for degradation (blunt arrow) or translocation (pointy arrow).

Rice (*Oryza sativa* L.), the staple food for more than half of the world’s population, is highly susceptible to abiotic stresses, such as drought, salinity, cold, high temperatures, nutrient deprivation, and toxicity (Lyzenga and Stone, 2012; Sharma et al., 2019). Several studies show that ubiquitination components and, in particular, monomeric E3-ubiquitin ligases are highly involved in rice response to different abiotic stresses. Therefore, in this review, we aim at describing and discussing the advances made on this subject, focusing on rice monomeric E3-ubiquitin ligases related to abiotic stress response (drought, salinity, and temperature stress; see Table 1 for a list of recently characterized rice E3-ubiquitin ligases, and Figure 1 for a schematic representation of their targets and effect on stress response) and prospects for their use in the development of stress-tolerant crops.

**TABLE 1:** Selected reported rice E3-ubiquitin ligases and their roles in abiotic stress responses.

E3 ligase	Type	Target	Stress tolerance	References
OsPUB67	U-box	OsRZFP34; OsDIS1	Drought (P)	Qin et al., 2020
OsDIS1	RING	OsNek6	Drought (N)	Ning et al., 2011a
OsCTR1	RING	OsBBTI4; OsMASP1; OsCP12; OsCYP450; OsDAHP1; OsDAHP1; OsXYLA1; OsENO1; OsRP1	Drought (P)	Lim et al., 2014
OsRDCP1	RING	unknown	Drought (P); Cold (I)	Bae et al., 2011
OsDSG1	RING	OsABI3	Drought (N); Salinity (N)	Park et al., 2010
OsSDIR1	RING	unknown	Drought (P); Salinity (I)	Gao et al., 2011
OsSIRP2	RING	OsTKL1	Salinity (P); Osmotic (P); Drought (I)	Chapagain et al., 2018
OsSADR1	RING	OsSNAC2; OsGRAS44; OsPIRIN	Salinity (N); Osmotic (N); Drought (I)	Park et al., 2018a
OsMAR1	RING	OsOCPI2	Salinity (N); Drought (I)	Park et al., 2018a
OsRMT1	RING	OsSalT; OsCPA1; OsbZIP60; OsFKBP12; OsEDA16; OsDH1; OsPUB53; OsPB1	Salinity (P)	Lim et al., 2015
OsSIRP1	RING	unknown	Salinity (N)	Hwang et al., 2016
OsRINGC2-1	RING	unknown	Salinity (P)	Jung et al., 2012
OsRINGC2-2	RING	unknown	Salinity (P)	Jung et al., 2012
OsSIRH2-14	RING	OsHKT2;1; OsSalT; OsPRF2	Salinity (P)	Park et al., 2019
OsPUB15	U-box	unknown	Salinity (P); Drought (I)	Park et al., 2011
OsPUB2	U-box	unknown	Cold (P)	Byun et al., 2017
OsPUB3	U-box	unknown	Cold (P)	Byun et al., 2017
OsHOS1	RING	OsICE1; OsEREBP1; OsEREBP2	Cold (N)	Lourenço et al., 2013, 2015
OsSRFP1	RING	Unknown; predicted	Cold (N); Salinity (N); Drought (I)	Fang et al., 2016
OsHIRP1	RING	OsAKR4; OsHKR1	Heat (P)	Kim et al., 2019
OsHTAS	RING	OsS27a; Os40SRPS; OsE2s	Heat (P)	Liu et al., 2016
OsDHSRP1	RING	OsGLYI-11.2; OsACP1	Heat (N); Drought (N); Salinity (N)	Kim et al., 2020
OsHCI1	RING	OsPGLU1; OsbHLH065; OsGRP1	Heat (P); Cold (I)	Lim et al., 2013

*P – positive regulation of stress response; N – negative regulation of stress response; I – induction by stress (see reference for further details).*

## E3-Ubiquitin Ligases in Drought Responses

It is estimated that more than 20 million hectares of rain-fed lowland rice (12% of the total rice area worldwide and about 20% of world’s production) are affected by drought at some point in the plant growth cycle (Zain et al., 2014; Ahmadikhah and Marufinia, 2016; FAO, 2019) with yield losses up to 81% (Ahmadikhah and Marufinia, 2016), and water scarcity events are predicted to worsen as extreme climate events become more frequent (Dixit et al., 2014). In rice, the effects of drought are especially harmful at the seedling stage (2 to 3 weeks old) and at the reproductive stage (pollen-development stage) (Dramé et al., 2013; Almeida et al., 2016), affecting flowering time and yield (Aroca, 2013). Overall, drought leads to poor water use efficiency, stomatal closure, impaired photosynthesis, and deficient cell division and expansion (Aroca, 2013). As a response strategy to cope with water deficit, plants modulate different genetic and metabolic mechanisms, such as cellular osmotic potential, stomatal aperture, antioxidant defense, phytohormones, and chlorophyll content, resulting in the adjustment and maintenance of their physiological activity under drought conditions (Fang and Xiong, 2015). Thus, it has become crucial to understand the molecular mechanisms underlying rice response to drought, namely the role played by ubiquitination, and to use this knowledge to develop tolerant crop varieties. During the last two decades, several rice E3-ubiquitin ligases and their interacting proteins have been associated with drought response in plants. Nonetheless, a more comprehensive analysis must be conducted to gain a deeper view of their interactome and function.

The U-box-domain containing E3-ubiquitin ligase OsPUB67 has recently been found to positively regulate drought tolerance by promoting enhanced scavenging of reactive oxygen species (ROS) and stomatal closure (Qin et al., 2020). *OsPUB67* is induced by different abiotic stresses (drought, salinity, and cold) and phytohormones, such as jasmonic acid (JA) and abscisic acid (ABA). At the seedling stage, *OsPUB67-*overexpressing lines showed an improved drought tolerance, underpinned by enhanced antioxidase activity, higher sensitivity to ABA, and decreased transpiration rate, leading to increased survival rates. In contrast, knockout lines showed higher sensitivity to drought and decreased survival rates under drought (Qin et al., 2020). It is known that drought triggers ABA accumulation leading to stomatal closure as a way of minimizing water loss (Aroca, 2013). In *OsPUB67-*overexpressing plants, a higher ratio of closed stomata under drought compared with the control condition was observed. This effect on stomatal control might be associated with OsPUB67 interaction with two targets (OsRZP34 and OsDIS1) identified by direct yeast-two hybrid (Y2H). OsRZFP34, which is localized in the membrane of guard cells of open stomata, was previously reported to play a negative role in stomatal closure and, consequently, decreasing drought tolerance. Indeed, overexpression of *OsRZFP34* in rice and Arabidopsis plants lead to an increased ratio of open stomata, even under ABA treatment (Hsu et al., 2014). Therefore, it can be hypothesized that, under drought conditions, OsPUB67 ubiquitinates and targets OsRZFP34 for proteolysis-mediated degradation, leading to the enhancement of stomatal closure. This hypothesis is further supported by the fact that the OsPUB67–OsRZFP34 interaction observed in the membrane of guard cells is weak under control conditions but becomes strong under drought when stomata closure is induced (Qin et al., 2020). OsPUB67 also interacts with *Oryza sativa* drought-induced SINA protein 1 (OsDIS1), another negative regulator of drought response in rice (Ning et al., 2011a). The OsPUB67–OsDIS1 interaction shows a pattern opposite to OsPUB67–OsRZP34’s, being weak in the membrane of closed stomatal guard cells and strong in the membrane of open stomata (Qin et al., 2020). Interestingly, OsRZP34 and OsDIS1 are also E3-ubiquitin ligases, which may account for another layer of complexity in the regulation of the response. Because the technique used to identify interactors (Y2H) was directed to a small number of proteins, it is possible that OsPUB67 targets other players important for the observed phenotype, but these were not yet identified.

OsDIS1 may play its negative role in drought stress response via the ubiquitination of a multitude of stress-related proteins (Ning et al., 2011a, b). Overexpression of *OsDIS1* in rice results in decreased drought tolerance, whereas the silencing of *OsDIS1* results in increased drought tolerance and survival rates (Ning et al., 2011a). OsDIS1 is predominantly localized in the nucleus but possibly translocates to the cytoplasm upon drought. Interestingly, one of its putative targets, *O. sativa* NIMA-related kinase (OsNEK6), is a microtubule (MT)-associated serine/threonine protein kinase, which is targeted for proteolysis via the proteasome. However, OsNEK6 has no known function in the rice response to stress. Given that MT are highly associated with stomatal movement (Qu et al., 2018) and OsPUB67–OsDIS1 interact in stomata guard cells, one may hypothesize that this module is important for the regulation of stomatal aperture under stress conditions. Further research is required to understand their mode of action. The rice homolog of human Ski-interacting protein (OsSKIPa), a positive regulator of drought and salt stress response in rice (Hou et al., 2009), is also shown to interact with OsDIS1 (2011b), and its accumulation seems to be regulated by the ubiquitin/26S proteasome system (UPS). Elsewhere, it is shown that *OsSKIPa* expression is induced by abiotic stresses, such as drought, salt, mannitol, and ABA, and its overexpression in rice leads to improved growth performance under the aforementioned stresses and ABA treatment. Conversely, the knockout of *OsSKIPa* in rice plants resulted in growth arrest and reduced cell viability (Hou et al., 2009). However, whether this degradation is promoted by OsDIS1-mediated ubiquitination or not remains to be solved.

*Oryza sativa chloroplast targeting 1* (*OsCTR1*) is another RING E3 ligase shown to be involved in water-deficit responses. Its gene expression is induced by dehydration and ABA treatment and, when overexpressed in Arabidopsis, confers drought tolerance (Lim et al., 2014). The Arabidopsis overexpression plants show ABA hypersensitivity, which can explain the observed enhanced tolerance and higher survival rates under severe water deficits. In control conditions, OsCTR1 localizes in both cytoplasm and chloroplast; however, under ABA treatment, OsCTR1 localizes mainly in the cytoplasm, where it interacts with several proteins although many of them are predicted to be chloroplast-based and related to photosynthesis (Lim et al., 2014). One of these interactors is the Calvin cycle protein CP12 (OsCP12), which is ubiquitinated by OsCTR1 in the cytoplasm and targeted for proteasome-mediated degradation (Lim et al., 2014). The Calvin cycle protein CP12 is involved in the regulation of the Calvin cycle and, hence, of sugar production (Wedel et al., 1997; Sugiura et al., 2019). In an oxidizing environment rich in H_2_O_2_, as, for instance, drought, CP12 is oxidized leading to a strong hampering of photosynthetic enzymatic activity (Yu J. et al., 2020). The ubiquitination-mediated degradation of OsCP12 could be a mechanism for guaranteeing the continuous production of energy supply even under drought. The results of this study indicate that OsCTR1 confers drought-tolerance possibly via an ABA-dependent pathway. It is essential to investigate the physiological role of OsCTR1 in rice as well as the mechanisms underlying drought tolerance. Do rice plants overexpressing OsCTR1 show an improved performance under drought? If yes, what are the molecular mechanisms underpinning this phenotype?

*Oryza sativa RING domain-containing protein 1* (*OsRDCP1*) is a RING E3-ubiquitin ligase upregulated by drought at a transcriptional level and whose overexpression enhances tolerance to severe water deficit in rice (Bae et al., 2011). Interestingly, loss-of-function plants showed a response to drought similar to WT. This phenotype may be due to a compensation effect by any of the other five *OsRDCP* paralogs present in rice. Interestingly, an OsRDCP1 homolog from *Capsicum annuum*, CaRma1H1, when overexpressed in Arabidopsis, also enhances drought tolerance (Lee et al., 2009). It is shown that *CaRma1H1* interacts with and inhibits the trafficking of the aquaporin PIP2;1 from endoplasmic reticulum (ER) to the plasma membrane by targeting it for degradation via the UPS. This indicates that the function on drought tolerance might be associated with the control of aquaporin levels in the plasma membrane. However, it is still necessary to fully characterize its physiological role in rice and identify its targets. It is important to produce knockout mutants (CRISPR/Cas9) for the five rice paralogs, including different combinations.

## E3-Ubiquitin Ligases in Salinity Responses

Similarly to drought, salinity is one of the top environmental factors affecting plant development and hindering crop yield (Hoang et al., 2016). It is estimated that 20% of the total global cultivated agricultural land is affected by high salinity with the affected area increasing every year (Shrivastava and Kumar, 2015). Rice is very sensitive to salt with most of its varieties having a salt electrical conductivity (EC) threshold as low as 3 dS m^–1^; subjecting rice to an EC of 6 dS m^–1^ would result in more than 50% reduction in rice grain yield (Lekklar et al., 2019). Therefore, it is imperative to better understand the molecular mechanisms underlying rice responses to high salinity and to identify new varieties capable of coping with the saline environment. During recent years, it has been shown that rice salt stress responses can be mediated by E3-ubiquitin ligases.

*Oryza sativa salt-induced RING finger protein 2* (*OsSIRP2*) is induced by high salinity, drought, and ABA and encodes a RING-type E3-ubiquitin ligase that localizes to the nucleus of rice protoplasts under both control and high-salinity conditions. *OsSIRP2*, when overexpressed in Arabidopsis, is shown to confer tolerance to salinity and osmotic stresses (Chapagain et al., 2018). It is also shown that OsSIRP2 is able to interact with the rice transketolase 1 (OsTKL1) in the cytoplasm, targeting it for degradation via the UPS. OsTKL1 belongs to the transketolase family involved in the oxidative pentose phosphate pathway of the Calvin cycle. It is essential for the regeneration of ribulose 1,5-bisphosphate (Murphy and Walker, 1982; Khozaei et al., 2015) and is localized in the chloroplast. The reduction of TKL1 activity leads to the inhibition of photosynthesis in tobacco (Henkes et al., 2001). Transketolase’s enzymatic activity is also crucial for the stress-induced production of cytosolic NADPH, a major component combating ROS-induced damage in a plant under stress (Tunc-Ozdemir et al., 2009). To better understand the function of OsSIRP2 and the physiological meaning of the OsSIRP2–OsTKL1 interaction in salt (and drought) stress responses, including photosynthesis performance, it is important to perform a functional characterization of these two proteins in rice. It is fundamental to understand if and how the negative regulation of OsTKL1 by OsSIRP2 promotes tolerance to stress. Finally, the translocation of OsSIRP2 from the nucleus to the cytoplasm to ubiquitinate OsTKL1 raises the question of the underlying mechanism driving this export because OsSIRP2 did not change localization under salt stress.

The RING-type E3-ubiquitin ligase encoded by the gene *Oryza sativa salt-, ABA-, and drought-induced RING finger protein 1 (OsSADR1)* is highly induced by salt, drought, and ABA treatments at the transcriptional level (Park et al., 2018a). Under non-stress conditions, OsSADR1 localizes in the cytosol and the nucleus, but upon salt stress, OsSADR1 accumulates in the nucleus. In addition, Arabidopsis plants overexpressing *OsSADR1* show increased sensitivity to salt, drought, and mannitol stresses, indicating that OsSADR1 acts as a negative regulator of abiotic stresses. This low tolerance to stress might be due to the ABA hyposensitive phenotype shown by these plants. OsSADR1 is shown to interact with the nuclear-localized stress-induced proteins OsSNAC2 and OsGRAS44 (GRAS family transcription factor domain-containing protein 44). OsSNAC2, a member of the stress-responsive NAM, ATAF, and CUC transcription factors family, is a positive regulator of salinity stress in rice and its overexpression in rice results in ABA hypersensitivity (Hu et al., 2008). The degradation of the OsSNAC2 Arabidopsis homolog mediated by OsSADR1 may be one of the reasons for the increased sensitivity to salt and other stresses. Further analysis of the molecular mechanisms underlying the OsSADR1 function should clarify its interaction with the ABA signaling pathway and the role of the interaction with the two targets, particularly OsGRAS44, in the response to abiotic stresses.

Park et al. (2018b) report that rice RING E3-ubiquitin ligase *MT associated RING finger protein 1* (*OsMAR1*) is highly upregulated by salinity and water deficit. However, the overexpression of *OsMAR1* in Arabidopsis results in lower tolerance to salt stress, indicating that OsMAR1 acts as a negative regulator of salt stress. In rice, OsMAR1 localizes to the MT but translocates to the cytosol, where it interacts with the cytosol-localized subtilisin-chymotrypsin protease inhibitor 2 (OsOCPI2) and targets it for degradation. OCPI2 is also induced by salt stress (Huang et al., 2007; Singh et al., 2009), but when overexpressed in Arabidopsis plants, improves salt tolerance (Tiwari et al., 2015). These results indicate that the interaction between the negative regulator OsMAR1 and the positive regulator OsOCPI2 play an important role in modulating salt stress response in rice. However, this hypothesis needs to be tested as the interaction between OsMAR1 and OsOCPI2 is so far only studied under control conditions. Furthermore, the functional role of OsMAR1 and OsOCPI2 remains to be validated in rice, and the localization of OsMAR1 to the MT should also be a subject of further study.

*Oryza sativa RING finger protein with MT-targeting domain 1* (*OsRMT1*) is another MT-associated E3-ubiquitin ligase shown to play a role in rice response to salinity stress (Lim et al., 2015). *OsRMT1* overexpression in Arabidopsis leads to an increased salt stress tolerance. Interestingly, under control conditions, OsRMT1 levels are tightly regulated by its own homodimer’s self-ubiquitination and degradation via UPS. However, under salinity, OsRMT1 is stabilized. The homodimeric OsRMT1 localizes to MT, where it also exerts its E3-ubiquitin ligase activity over most of its identified targets. Interestingly, all the target proteins identified (OsSalT, OsCPA1, OsbZIP60, OsFKBP12, OsEDA16, OsDH1, OsPUB53, and OsPB1) had their transcripts highly upregulated in salt imposition and were reported to be salt- or osmotic stress-responsive (Claes et al., 1990; Ahn et al., 2010; Zhang et al., 2012; Chung et al., 2018; Pandey et al., 2018; Liu et al., 2020). OsRMT1, which is also found in the nucleus, translocates the target proteins to the MT. This mode of action of an MT-localized E3-ubiquitin ligase is different from that of the abovementioned E3-ubiquitin ligase OsMAR1, which translocates to the cytosol to exert its ubiquitin ligase activity (Park et al., 2018b). However, the physiological function of OsRMT1 in rice remains to be investigated.

OsRMT1 and OsMAR1 studies shed new light on the regulation of E3-ubiquitin ligases and raises the question about the mode of action of different types of E3-ubiquitin ligases associated with the cytoskeleton. A subsequent step in the analysis of these E3-ubiquitin ligases might be the possible effect of the target proteins in the reorganization of the cytoskeleton upon stress.

## E3-Ubiquitin Ligases in Temperature Stress Responses

Temperature stress, such as cold and heat, hinder plant development and growth by affecting water potential, ROS, phytohormones, photosynthesis performance, fertility, and crop yield and quality (da Cruz et al., 2013; Kilasi et al., 2018; Liu et al., 2018). Rice is a very sensitive crop to both cold and heat stresses (Kilasi et al., 2018; De Freitas et al., 2019). Indeed, it has been reported that the critical temperature for spikelet fertility in rice is a maximum of 35°C (Wang et al., 2019). Moreover, sustained high temperatures for more than a week cause severe heat injury (Wang et al., 2019). Similarly, sustained low temperatures cause cold injury and sterility (Liu et al., 2013). Therefore, it is of paramount importance to better understand the molecular mechanisms underlying rice responses to low and high temperatures.

### Response to Low Temperature

The homologous U-box type E3-ubiquitin ligase proteins OsPUB2 and OsPUB3 were recently characterized as positive regulators of cold stress response in rice (Byun et al., 2017). *OsPUB2* is upregulated by low temperature, drought, and high salinity, and *OsPUB3* expression does not respond to any of the aforementioned stresses. However, overexpression of either *OsPUB2* or *OsPUB3* in rice plants confers a cold-tolerance phenotype, including enhanced survival rates, increased chlorophyll content, and reduced ion leakage. Furthermore, gene expression analysis shows that the overexpression of the two *OsPUB* genes is associated with the upregulation of cold stress inducible genes, such as *glutamate decarboxylase* (*GAD*), *WRKY77*, and *multidrug resistance protein 4* (*MRP4*), under both control and cold conditions, and *trehalose-6-phosphatephosphatase 2* (*TPP2*) and *MYBS3* were induced only under drought conditions, and *DREB1B/CBF1* decreased under cold stress compared with wild-type plants. *RNAi* knockdown rice plants show a phenotype similar to WT; however, these results may be due to the redundant function of OsPUB2 and OsPUB3. This hypothesis could be tested by performing a simultaneous full knockout of both genes. However, the mode of function of these proteins may provide a clue for the knockout mutant phenotype. The two E3-ubiquitin ligases, which share a high degree of sequence identity (75%), exert their ubiquitin ligase activity by forming homodimer and heterodimer complexes, with the latter being more stable. Moreover, both had their stability enhanced by cold. The subcellular localization in *Nicotiana benthamiana* leaf protoplasts shows both E3-ligases in small cytosolic punctate bodies. Because OsPUB2 was also observed in the nucleus, it raises the possibility of being involved in an additional process, different from its homolog. It remains, however, to be shown whether those two homologous E3-ubiquitin ligases work together to confer cold tolerance to rice plants and by means of what target protein(s) this tolerance is achieved. Moreover, the OsPUB2 mutants should be analyzed under multiple stresses for further phenotypic analysis.

Another E3-ubiquitin ligase functioning as a modulator of plant response to cold stress in rice is the RING-type high expression of osmotically responsive gene 1 (*OsHOS1)* (Lourenço et al., 2013). OsHOS1 interacts with Inducer of CBF expression 1 (OsICE1) in the nucleus and targets it for degradation via the UPS. *OsHOS1*-silenced (RNAi) lines show higher transcript levels of the stress-responsive transcription factor *dehydration-responsive element (DRE)-binding protein 1A* (*OsDREB1A*) and protein levels of OsICE1, a master integrator of cold stress signaling regulating the expression of cold-responsive genes (Lourenço et al., 2013). However, the higher levels of *OsDREB1A* are transient and do not confer cold tolerance or enhanced survival rates to the RNAi lines. This role is also observed for the OsHOS1 ortholog in Arabidopsis (HOS1) in response to cold stress (Dong et al., 2006). OsICE1, also known as OsbHLH002, is a positive regulator of cold stress response in rice. It is shown that, when cold stress is applied only in roots, the overexpression of OsICE1 confers cold tolerance to the plant (Zhang et al., 2017). Conversely, knockout of OsICE1 results in cold hypersensitivity. Furthermore, it was found that, under cold stress, mitogen-activated protein kinase 3 (OsMAPK3) phosphorylates OsICE1, leading to its accumulation and protection against ubiquitination by OsHOS1 (Zhang et al., 2017). Interestingly, but not surprisingly, phosphorylation of OsICE1 by OsMAPK3 takes place in the nucleus, where OsICE1 ubiquitination also occurs. Evidence suggests that, in warmer temperatures, OsICE1 levels are maintained via the UPS system, whereas under cold, this regulation is disrupted by OsMAPK3, leading to OsICE1 stabilization and activity enhancement (Zhang et al., 2017). However, further studies are needed to unveil how this state change takes place and the effect on the full plant life cycle. Interestingly, OsHOS1 is also found to be involved in rice root mechano-sensing through JA signaling by targeting two rice EREBP TFs for degradation (Lourenço et al., 2015). Whether these targets and JA are involved in OsHOS1-mediated cold response in this species remains to be elucidated.

The *Oryza sativa* stress-related RING finger protein 1 (OsSRFP1) is an unusual E3-ubiquitin ligase that shows both transcriptional and post-translational activity in plant response to cold stress (Fang et al., 2015, 2016). The *OsSRFP1* expression level is induced by cold, PEG-simulated drought, salt, H_2_O_2_, and ABA, and its overexpression in rice results in a hypersensitive phenotype and yield penalty although the opposite is observed for RNAi silencing lines under cold stress conditions. The silencing lines show increased levels of proline, higher activity of antioxidant enzymes, and increased growth and survival rates. On the other hand, overexpression plants show decreased levels of proline and lower activity of the antioxidant enzymes. Interestingly, the transcript profile of the *OsSRFP1* overexpression plants reveals that many genes involved in ROS homeostasis are downregulated under cold stress, suggesting that the negative effect of OsSRFP1 under cold stress is achieved by modulating the expression of such genes. OsSRFP1 shows a dual localization, being partitioned between the nucleus (predominantly) and the cytoplasm. OsSRFP1 exhibits transcriptional activity in both yeast and rice protoplasts, assessed by the ability of the (BD)-OsSRFP1 fusion protein to activate the transcription of the GAL4 gene with the RING domain being essential for this activity (Fang et al., 2015). *In silico* prediction of the *cis*-acting elements for OsSRFP1 includes ABRE, NACF, EPFF, and MYBS, and putative interactors are several members of the MYB family of transcription factors. However, all lack experimental validation. The OsSRFP1 homolog in apple, *Malus domestica* MYB30-Interacting E3 Ligase 1 (MdMIEL1), is also shown to respond to and/or regulate several stresses and auxin accumulation (An et al., 2017, 2020). *MdMIEL1* overexpression in Arabidopsis results in lower cold tolerance, and similar to rice, the antisense transgenic plants showed an enhanced performance under cold stress. MdMIEL1 exerts its negative role in plant response to cold stress by interacting with and targeting for degradation the positive regulator of cold stress MdMYB308L, an MYB-type transcription factor (An et al., 2020). Given this evidence, and to better characterize the cold-stress response mechanism exerted by OsSRFP1, further studies are needed to unveil the targets of OsSRFP1.

### Response to Heat Stress

*Oryza sativa* heat-induced RING finger protein 1 (OsHIRP1) is a RING-type E3-ubiquitin ligase shown to act as a positive regulator of heat stress in Arabidopsis (Kim et al., 2019). The overexpression of *OsHIRP1* in Arabidopsis leads to a high germination and survival rates under heat stress. In rice protoplasts under control conditions, OsHIRP1 localizes to both cytosol and nucleus; however, upon heat stress (45°C) treatment, the enzyme is located predominantly in the nucleus. OsHIRP1 interacts directly with the putative aldo/keto reductase family protein 4 (OsAKR4) in the nucleus and with the OsHIRP1-regulated Kinase 1 (OsHRK1) in the cytosol. OsHIRP1 directly targets OsAKR4 and OsHRK1 for degradation via the UPS under heat stress (45°C) but not at control or low temperatures (4°C), indicating that degradation of those target proteins via UPS is temperature dependent. The OsAKR4 Arabidopsis ortholog, potassium channel beta subunit 1 (KAB1), also possesses an aldo/keto reductase motif and is a voltage-gated protein involved in the inward transport of potassium in the guard cells, thus contributing to stomatal opening (Sharma et al., 2013). It is, therefore, possible that OsAKR4 degradation is a means of avoiding water loss (Ilan et al., 1995). However, the overexpression of *OsAKR4* in rice is shown to enhance tolerance to high temperatures (Kim et al., 2019). It is likely that, under high temperatures, OsAKR4’s effect on stomatal opening is important for leaf cooling, but at a certain point, it is also important to balance this to avoid exaggerated water loss. Its protein abundance may vary along heat stress, and its regulation by OsHIRP1 might play an important role regulating stomatal aperture. It is essential to further investigate this to decipher the mechanism governing the location of OsHIRP1 interaction with its targets as well as unveil the biological meaning of OsHRK1.

The E3-ubiquitin ligase *Oryza sativa* drought-, heat-, and salt-induced RING finger protein 1 (OsDHSRP1) is shown to be highly induced by salinity, heat, and drought at the transcript level. In addition, it is shown in Arabidopsis to act as a negative regulator of various stresses, especially heat stress (45°C), modulating the abundance of stress-responsive proteins via the UPS (Kim et al., 2020). The overexpression of *OsDHSRP1* in *Arabidopsis* leads to hypersensitivity to heat stress. OsDHSRP1 is shown to be associated with MT; however, it interacts with, ubiquitinates, and targets for degradation a glyoxalase (OsGLYI-11.2) and the abiotic stress-induced cysteine proteinase 1 (OsACP1) elsewhere in the cytoplasm. This raises the question about the molecular mechanism behind the difference in the localization of OsDHSRP1 as well as that of its interaction. OsDHSRP1 is also shown to act in the same way with the rice target Arabidopsis homologs (AtGLYI-11.2 and AtACP1, respectively). OsGLYI-11.2 is a member of the glyoxalase system, which is responsible for the detoxification of methylglyoxal (MG), a cytotoxic byproduct of metabolism. Levels of MG are shown to increase under abiotic stress, leading to adverse effects in plants (Mustafiz et al., 2014). In addition, it is known that overexpression of glyoxalases in plants confers tolerance to multiple abiotic stresses (Belavadi et al., 2017). Nevertheless, the role of OsGLYI-11.2 in rice response to heat stress needs to be more deeply investigated. OsACP1 belongs to the cysteine proteinase group, which represents the majority of proteolytic activity in plants. OsACP1 is induced by methyl jasmonate, salicylic acid, salt, and heat stress but not by drought. Furthermore, *OsACP1* overexpression appears to confer salt tolerance in rice (Niño et al., 2020). Still, further characterization of OsACP1’s role in rice response to heat is required to understand the underlying mechanism. It would also be important to carry out a detailed functional characterization of OsDHSRP1 in rice and identify new interactors specifically involved in the heat stress responses.

*Oryza sativa* heat- and cold-induced 1 (*OsHCI1*) is a RING E3-ubiquitin ligase highly induced under extreme temperatures (4 and 45°C) but not by salinity or dehydration stresses and, when overexpressed in Arabidopsis, confers high tolerance to heat stress (Lim et al., 2013). Under control conditions, OsHCI1 localizes mostly in the cytoplasm, being partitioned between the cytoplasm and the nucleus during heat stress. Under heat stress, OsHCI1 moves from the Golgi complex, where it is mainly localized, to the nucleus via the cytoskeleton (Lim et al., 2013). In the nucleus, OsHCI1 monoubiquitinates and promotes the nuclear export of its nuclear-localized target proteins to the cytoplasm; these include a periplasmic beta-glucosidase (OsPGLU1), a bHLH transcription factor (OsbHLH065), and a glycine-rich cell-wall structural protein (OsGRP1), which are nuclear-localized under control conditions (Lim et al., 2013). OsPGLU1 belongs to the class of beta-glucosidases enzymes that catalyze the hydrolysis of oligosaccharides from the plant cell wall into glucose. Beta-glucosidases have been associated with several biotic and abiotic stress responses, including cold (Thorlby et al., 2004; Cairns and Esen, 2010). However, a role for OsPGLU1 in plant response to abiotic stress has not yet been reported. Nevertheless, the maintenance of its transcripts at a high level throughout the heat treatment (Lim et al., 2013) may indicate that such action is an adaptive mechanism to heat stress, namely via the alteration of sugar metabolism as an alternative source of energy to the diminishing photosynthesis (Rouyi et al., 2014). OsGRP1, also known as glycine-rich RNA-binding protein (Osgr-rbp4), is an mRNA regulator and is reported as conferring tolerance to heat stress in yeast and to be translocated from the nucleus to the cytoplasm upon heat shock (Sahi et al., 2007). Under current evidence, the translocation of OsPGLU1 and OsGRP1 from the nucleus to the cytoplasm promoted by OsHCI1 via monoubiquitination represents a non-proteolytic function of this enzyme on an abiotic stress condition. Whether OsHCI1 overexpression in rice also improves heat tolerance and, if so, whether this phenotype is associated with its interaction with the proteins identified, remains to be investigated.

## Concluding Remarks

Ubiquitination is considered to be a major form of post-translational modification, modulating plant growth and development, and a key component of the plant response to abiotic stresses (Vierstra, 2009, 2012; Callis, 2014). The E3-ubiquitin ligase has been singled out as the major determinant of target protein fate and, ultimately, the physiological effect thereof (Shu and Yang, 2017). As shown in this review, the E3-ubiquitin ligases act either as positive or negative regulators of abiotic stress responses. Furthermore, the positive or negative role of E3-ubiquitin ligases in response to abiotic stresses depend on the protein being targeted and the result of the modification thereof, namely UPS-mediated degradation, modulation of activity, or translocation. Therefore, the identification and characterization of ubiquitin ligase targets should constitute a central point in any stress response study.

E3-ubiquitin ligases often appear to be associated with MT. The E3-ubiquitin ligase role in MT regulation seems to be transversal to cellular processes and to species, such as hypocotyl elongation (Lian et al., 2017), formation of mammalian spermatozoa (Iyengar et al., 2011), cell migration (Courtheoux et al., 2016), cell division (Maerki et al., 2009; Venuto and Merla, 2019), and viral infection (Liu et al., 2010). The MT role in stress response is not surprising given that MT play a sensory role in the perception and response of mechanical stress and other abiotic stresses, such as cold, heat, and osmotic stress (Nick, 2013). In *Arabidopsis*, salt stress leads to the UPS-dependent degradation of MT-associated protein SPIRAL1 (SPR1), which is responsible for maintaining MT stability. Degradation of SPR1 results in the depolymerization of MTs followed by the formation of new MTs better adapted to osmotic stress (Wang et al., 2011). Another example is the E3-ubiquitin ligase JAV1-associated ubiquitin ligase 1 (JUL1), which, in Arabidopsis, localizes to the MTs and is thought to be crucial for ABA-mediated regulation of stomatal closure under drought stress conditions by means of depolymerization of MTs in the guard cells (Yu S. G. et al., 2020). The MT-associated rice E3-ubiquitin ligases mentioned in this review, OsMAR1, OsRMT1, and OsDHSRP1, are shown to be involved in salinity, heat, and drought. However, these rice E3-ubiquitin ligases have not yet been investigated for their potential role in MT dynamics. Therefore, it can be hypothesized that some MT-associated E3-ubiquitin ligases may function to mediate MT remodeling in response to internal or external environmental stimuli.

**FIGURE 2:**
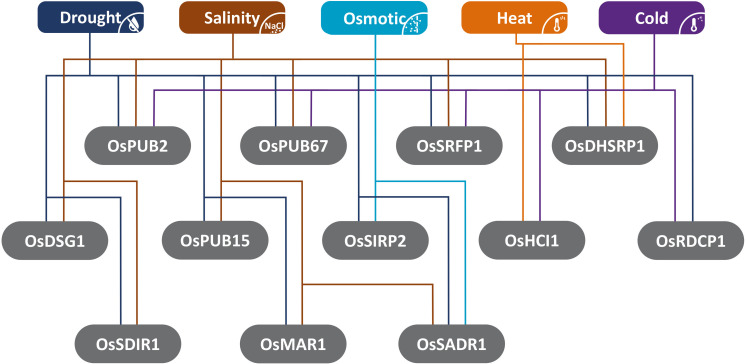
Illustration summarizing the different abiotic stress responses of rice E3-ubiquitin ligases described in this review.

Many of the studied E3-ubiquitin ligases respond to a multitude of stresses and may mediate the crosstalk between stresses (Figure 2), such as drought, salinity, pathogens, nutrient deprivation, and toxicity (Stone, 2014; Shu and Yang, 2017). It is reported that the combination of stresses has different and unique effects on plant physiology and cannot be deduced from individual stresses (Suzuki et al., 2016). Therefore, it is crucial that more studies on the ubiquitination pathway tap into the nature of ubiquitination-mediated plant response to combined abiotic stresses.

It is extensively reported that transgenic plants achieve a stress-tolerant phenotype, either by overexpressing or knocking down/out stress-responsive E3-ubiquitin ligases. This opens up the prospect of the use of genetic modification tools, such as CRISPR/Cas9, for the production of E3 ligase transgenic crops better suited for stress-prone environments. However, the study of the ubiquitination pathway still faces many challenges that may be addressed in future studies to move forward in this field. For instance, the study of membrane E3-ubiquitin ligases poses a particular challenge. The study of interactomes through standard techniques, such as Y2H or bimolecular fluorescence complementation (BiFC), cannot be readily achieved. This is especially because, in Y2H, the protein must be shuttled to the nucleus, which does not happen with membrane proteins, and in BiFC, the fluorescence fusion tag may hinder its structure and, therefore, limit binding. Nevertheless, alternative and complementary methods, such as the split-ubiquitin system (Johnsson and Varshavsky, 1994), proximity labeling (Roux et al., 2012), and ratiometric BiFC (rBiFC) (Grefen and Blatt, 2012) have been develop and prove very useful to address the challenge of uncovering E3-ubiquitin ligase interactomes.

An additional challenge relates to the understanding of the formation of the ubiquitin chain. As mentioned, the type of ubiquitination results in different fates for the target protein. The number of ubiquitination sites in the target protein combined with the type and length of the ubiquitin chain, result in an enormous number of possibilities regarding the fate of the modified target proteins. However, the study of this ubiquitin system versatility is restricted by the available tools. Techniques such as mass spectrometry enable mapping of ubiquitination modifications; however, only recently the development of the Ub-clipping methodology has allowed for an in-depth look at the polyubiquitin chain signals and architecture, as well as coexisting ubiquitin modifications (Swatek et al., 2019). Besides the type of ubiquitin chain added to a target protein, the removal of ubiquitin or a ubiquitin chain by DUBs is also a promising, underexplored field of research. Previous work in Arabidopsis shows that DUBs are involved in several developmental processes as well as phytohormone and stress responses (Zhou et al., 2013; Zhao et al., 2016; Jeong et al., 2017; March and Farrona, 2018). In rice, so far, very few DUBs have been functionally characterized, and none directly involved, in abiotic stress responses (Moon et al., 2009; Wang et al., 2017). A brief *in silico* analysis of putative rice DUBs have shown consistent gene expression changes in response to abiotic stress (data not shown). Therefore, it will be extremely interesting to follow the DUB counterparts of E3-ubiquitin ligases to have a comprehensive understanding of the ubiquitin pathway function in abiotic stress responses.

The recent finding that the variation of a single a.a. in an E3-ubiquitin ligase target protein may have contributed to the geographic variation in heading date among *japonica* rice accessions, i.e., differential rice adaptation to different environments, opens up new prospects for the search for natural variability either in E3-ubiquitin ligases or in the target proteins (Zhu et al., 2018) in rice and other crops. In rice, although more than 3000 genomes are available (Sun et al., 2016) and SNPs identified, the phenotypic characterization under stress is still very scarce. This is, however, a very promising source of information that must be exploited to identify natural variability of the rice E3 ligases and, thus, unveil new tools for crop improvement through traditional or new breeding technologies.

The study of ubiquitin ligases in crops, compared with other plants such as Arabidopsis, remain subpar; however, the prospect of development of ubiquitination-based stress-tolerant crops has been steadily increasing during the past decade. With an increasing interest in this field and the new technologies available, we may soon see results in crop agronomic performance.

## Author Contributions

FM, NS, and TL drafted the review. FM prepared this manuscript and produced Figures 1, 2 and Table 1. MO, NS, and TL reviewed the manuscript, Figures 1, 2 and Table 1. All authors agreed on the final version of the manuscript.

## Conflict of Interest

The authors declare that the research was conducted in the absence of any commercial or financial relationships that could be construed as a potential conflict of interest.
